# Clinical Perspectives on Liquid Biopsy in Metastatic Colorectal Cancer

**DOI:** 10.3389/fgene.2021.634642

**Published:** 2021-01-28

**Authors:** Wei Gao, Yigui Chen, Jianwei Yang, Changhua Zhuo, Sha Huang, Hui Zhang, Yi Shi

**Affiliations:** ^1^Department of Internal Medicine-Oncology, Fujian Cancer Hospital and Fujian Medical University Cancer Hospital, Fuzhou, China; ^2^Department of Gastrointestinal Surgical Oncology, Fujian Cancer Hospital and Fujian Medical University Cancer Hospital, Fuzhou, China; ^3^Department of Hepatopancreatobiliary Surgical Oncology, Fujian Cancer Hospital and Fujian Medical University Cancer Hospital, Fuzhou, China; ^4^Department of Molecular Pathology, Fujian Cancer Hospital and Fujian Medical University Cancer Hospital, Fuzhou, China

**Keywords:** metastatic colorectal cancer, liquid biopsy, biomarker, prediction, therapy response

## Abstract

Liquid biopsy, which generally refers to the analysis of biological components such as circulating nuclear acids and circulating tumor cells in body fluids, particularly in peripheral blood, has shown good capacity to overcome several limitations faced by conventional tissue biopsies. Emerging evidence in recent decades has confirmed the promising role of liquid biopsy in the clinical management of various cancers, including colorectal cancer, which is one of the most prevalent cancers and the second leading cause of cancer-related deaths worldwide. Despite the challenges and poor clinical outcomes, patients with metastatic colorectal cancer can expect potential clinical benefits with liquid biopsy. Therefore, in this review, we focus on the clinical prospects of liquid biopsy in metastatic colorectal cancer, specifically with regard to the recently discovered various biomarkers identified on liquid biopsy. These biomarkers have been shown to be potentially useful in multiple aspects of metastatic colorectal cancer, such as auxiliary diagnosis of metastasis, prognosis prediction, and monitoring of therapy response.

## Introduction

One of the most prevalent cancers worldwide, colorectal cancer (CRC) is the second leading cause of cancer-related mortality (Bray et al., 2018). Metastatic CRC constitutes an inevitable challenge for clinical management because of the considerably worse survival of metastatic patients compared to patients with non-metastatic CRC (Dekker et al., 2019). Approximately one quarter of CRC patients have metastasis when they are diagnosed and a considerable number of non-metastatic patients postoperatively develop metastasis. Eventually, more than half of all CRC patients will progress to metastatic disease and require corresponding therapy to prolong survival (Labianca et al., 2013; Siegel et al., 2020). Thus, early detection of CRC patients at high risk of developing metastatic CRC is crucial for early intervention to improve patient outcomes and save treatment costs. Current therapies for metastatic CRC patients include surgical resection, chemotherapy, targeted therapy, and immunotherapy (Woo and Jung, 2017; Chakedis and Schmidt, 2018; Nappi et al., 2018; Wrobel and Ahmed, 2019). The individualized selection and sequence of therapies usually differ noticeably based on findings on multidisciplinary evaluation of CRC patients. Therefore, biomarkers, including diagnostic, prognostic, and predictive factors obtained from cancer biopsies are critically important for guiding the therapeutic strategy for patients with metastatic CRC.

Owing to the intratumoral heterogeneity and dynamics of cancer genome modifications of the treatment or the development of cancer (Yates and Campbell, 2012; McGranahan and Swanton, 2015), routine tissue biopsies have a limited capacity to comprehensively obtain real-time information. There is an urgent need for a biopsy method without the disadvantages of tissue biopsy such as patient discomfort, risk of tumor seeding, limited sample accessibility, and procedural complications. The development of precision medicine could potentially eliminate the abovementioned limitations by liquid biopsy, which generally refers to the testing of biological components obtained from body fluids, especially whole blood. Several recent excellent reviews have discussed the application and potential scenarios of liquid biopsy in CRC (Gargalionis and Papavassiliou, 2017; Klein-Scory et al., 2018; Normanno et al., 2018; Tarazona and Cervantes, 2018; Wills et al., 2018; Yamada et al., 2019; Ding et al., 2020). Herein, we focus on the current application and clinical perspectives of liquid biopsy in metastatic CRC, particularly on advances in the discovery of potential roles of liquid biopsy in diagnosing metastasis, prognosis assessment, and therapy response monitoring.

## Methods of Liquid Biopsy

The analytes of liquid biopsy mainly refer to circulating tumor DNA (ctDNA), circulating tumor cells (CTC), and circulating non-coding RNAs, which include non-coding RNAs released by cancer cells into the circulation and those traveling inside exosomes (Poulet et al., 2019; Rapado-González et al., 2019). Following a brief introduction of each analyte, their performance consistency with tissue biopsy, especially in metastatic CRC, is reviewed and compared in this section.

The fragmented DNA released by tumor cells into the circulating system constitute CtDNA, which carry the genetic information of both primary-site and metastatic-site tumors, and are mainly detected by PCR and next-generation sequencing (NGS) in liquid biopsy. The most sensitive PCR-based methods (e.g., digital PCR) detect unique hotspots or well-identified mutations, whereas NGS-based methods have greater advantage in broad-range screening of mutations (Franczak et al., 2019). Several studies that evaluated the concordance between the detection of plasma-based ctDNA and tissue biopsy-based genomic DNA in metastatic CRC confirmed a high overall agreement (Grasselli et al., 2017; Bando et al., 2019; Galbiati et al., 2019; Kang et al., 2020; Yu et al., 2020). A prospective retrospective cohort study that enrolled 146 metastatic CRC patients compared the *RAS* mutational status by the plasma ctDNA-BEAMing (digital PCR) and tissue reference methods, and showed an 89.7% concordance rate (Grasselli et al., 2017). A similar agreement of 86.4% was recently demonstrated in another multicenter prospective study of 280 patients with metastatic CRC (Bando et al., 2019). A higher concordance rate of 92% between plasma ctDNA detected by digital PCR and tissue reference DNA was identified in common *KRAS* and *BRAF* mutations among 150 patients with metastatic CRC (Yu et al., 2020). However, another study reported a low concordance of 63.3% for the *KRAS* gene, which was explained by the different timings of liquid and tissue biopsies of the patients (Galbiati et al., 2019), and highlights the distinct mutational profiles among patients with different cancer stages. Conversely, ctDNA detected by NGS and tumor genomic DNA showed high agreement. Using ultra-deep target sequencing, Kang et al. (2020) investigated mutations in 10 genes (38-kb length) from ctDNA and genomic DNA derived from matched tumor tissues, and found an overall 93% concordance rate.

Primary tumors or metastases shed CTC that circulate in peripheral blood and can be isolated by various methods based on epithelial markers expressed on the cell surface or on the physiological properties of cells (Pantel and Speicher, 2016; Cabel et al., 2017). The detection capacities of CTC and ctDNA for metastatic CRC were, respectively, evaluated in parallel in a cohort of 20 patients; ctDNA could be detected in all patients with metastatic CRC, whereas CTC were detectable in only one third of the patients (Germano et al., 2018). Buim et al. (2015) analyzed KRAS mutations in both CTC and matched primary tumor samples, and observed a concordance rate of 71% between CTC and tissues. Another study showed 50% *KRAS* mutation-status agreement between CTC and matched primary tumors of patients with metastatic colon cancer (Fabbri et al., 2013).

Studies have indicated that, besides ctDNA and CTC, circulating non-coding RNAs are emerging as important biomarkers for the clinical management of CRC patients (Rapado-González et al., 2019; Baassiri et al., 2020). In body fluids, circulating non-coding RNA mainly exist in either the cell-free state or the exosomal form. Exosomes are nano-sized extracellular vesicles secreted by various cell types and can be isolated using ultracentrifugation, density-based separation, and antibody-based immune-affinity capture (Greening et al., 2015). Furthermore, the intra-exosomal expression levels of non-coding RNAs could be evaluated. In a study of 326 CRC patients, the expression levels of exosomal miR-21 significantly correlated with those of the CRC tissue miR-21 (Tsukamoto et al., 2017). Similar correlations have been reported for the expression levels of miR-122 (Sun et al., 2020), miR-25-3p (Zeng et al., 2018), etc. The strengths and limitations of the abovementioned approaches are shown in Table 1 (Jia et al., 2017; Nordgård et al., 2018; Normanno et al., 2018; Burz and Rosca, 2019; Pantel and Alix-Panabières, 2019).

**TABLE 1:** Strengths and limitations of applying ctDNA, CTC, and non-coding RNA (Jia et al., 2017; Nordgård et al., 2018; Normanno et al., 2018; Burz and Rosca, 2019; Pantel and Alix-Panabières, 2019).

	**Strengths**	**Limitations**
ctDNA	Well-established methods (e.g., NGS, ddPCR, ARMS, BEAMing, etc.) for detection and data analysis High sensitivity Identifying molecular and genomic features (e.g., bMSI, bTMB, DNA methylation) Analyzing cancer origin Predicting drug effects and acquired resistance Postoperative detection of minimal residual disease Monitoring metastatic process and disease progression	Discrimination of ctDNA in ctDNA pool Very low ctDNA abundance Impossibility of functional assays Impossibility of RNA and protein analysis Limited methodological standardization False-positives in certain high-sensitivity methods False-negatives due to the extremely low ctDNA level or interindividual variations of ctDNA abundance
CTC	Identifying morphological and molecular features Analyzing DNA, RNA, and protein profiles Functional assays *in vitro* or *in vivo* Analyzing cancer origin Utility as a prognostic or predictive marker Monitoring metastatic process and disease progression	Low CTC abundance CTC fragility Heterogeneity of CTC populations Limited methodological standardization False-positives due to normal cells from gastrointestinal inflammatory diseases False-negatives due to the difficulty of CTC isolation and tumor metastasis
Circulating non-coding RNA	Analyzing RNA expression profiles Potential marker for diagnosis, prognostic, and prediction	Low abundance and instability Difficult extraction Limited methodological standardization Requirement for more clinical evidences

*ctDNA, circulating tumor DNA; CTC, circulating tumor cells.*

### Roles of Liquid Biopsy in Metastatic Crc

The clear advantages of liquid biopsy, compared with routine tissue-based methods, have generated interest in the application of non-invasive hematological methods to clinical oncology that has steadily increased in the past decades. From the clinical perspective of metastatic CRC, multiple aspects of liquid biopsy, primarily for the identification of diagnostic roles for metastasis, prognostic biomarkers, and monitoring the therapy response, have been explored.

#### Auxiliary Staging: Metastasis Diagnosis

Apart from the driver mutations shared with the primary tumor, metastatic cancers often carry new mutations, which may play essential roles in the metastatic process (Turajlic and Swanton, 2016; Robinson et al., 2017). The molecular landscape of metastatic tumors, rather than that of exclusive primary tumors, is preferably used to guide potential therapies or clinical trials for patients with metastatic cancer (Steeg, 2016). A review recently summarized the potential and methods of tumor metastasis prediction from genome sequencing data using various tools based on machine learning, protein–network, or biological pathways (Yuan et al., 2019). Liquid biopsy is capable of detecting emerging mutations in metastatic CRC. A study of 22 CRC patients with liver metastases compared NGS and digital PCR detection [sensitivity of 64% (23/36) and 89% (32/36)] of metastasis-related mutations in peripheral blood samples (Furuki et al., 2018). Another study conducted whole-exome sequencing of ctDNA in plasma samples, and used “differential presence of exon (DPE) analysis” to distinguish between metastatic and non-metastatic CRC, and the results imply that DPE characteristics might have a diagnostic value for CRC patients (Olmedillas-López et al., 2018). The association between the ctDNA level and CRC stage has been studied. Yang et al. (2018) analyzed ctDNA levels in 47 CRC patients in early or late cancer stages and found that Stage IV patients had significantly higher ctDNA concentrations than Stage I patients. These studies support the implication that ctDNA characteristics could facilitate the diagnosis of metastatic CRC.

The CTC are well known to play important roles in tumor metastasis (Massagué and Obenauf, 2016; Dasgupta et al., 2017; Micalizzi et al., 2017). Based 2 years of follow-up data and a microfluidic device utilizing antibody-conjugated non-fouling coating to capture and enumerate CTC, a multicohort study that included healthy control, non-metastatic, and metastatic CRC patients explored the correlation between neoplasm progression and CTC, and showed that a high CTC count was significantly associated with tumor progression, metastasis, and future occurrence of distant metastases in non-metastatic CRC patients (Tsai et al., 2016). An earlier study showed that liver metastasis in CRC patients was associated with apoptotic CTC, instead of intact CTC, in the peripheral blood (Allen et al., 2014).

Non-coding RNAs, especially the more studied microRNAs, are involved in tumor cell invasion, migration, and progression in CRC (Cekaite et al., 2016). The expression of circulating non-coding RNA generally reflects the profiles of both primary tumor and metastatic lesions, and non-coding RNA has potential application as a biomarker for the detection of metastatic cancer. Therefore, a study of CRC patients with or without liver metastases showed that the serum miR-29a expression level could, with 75% sensitivity and specificity, help to differentiate between patients with metastatic and non-metastatic CRC (Wang and Gu, 2012). A high serum miR-200c level was significantly associated with metastasis in CRC (Toiyama et al., 2014), and the serum miR-203 level significantly increased in relation to the tumor stage, especially in patients with liver or systemic metastasis (Hur et al., 2017). These results suggest the potential role of these serum microRNAs as metastasis-predictive biomarkers in CRC.

Besides the abovementioned cell-free microRNAs, potential roles of exosomal microRNAs in diagnosing metastasis have been extensively investigated. For example, the high expression of serum exosomal miR-203 correlated with distant metastasis, and xenograft mouse experiments further showed that miR-203-transfected CRC cells had more liver metastasis than controls (Takano et al., 2017). Exosomal miR-25-3p levels were significantly higher in metastatic CRC than in non-metastatic CRC (Zeng et al., 2018). Screening of differential exosomal microRNAs by sequencing and qPCR showed that exosomal miR-320d could distinguish, with an area under the ROC curve (AUC) of 0.633, a diagnosis of metastatic CRC (Tang et al., 2019). The expression of exosomal miR-139-3p was significantly decreased in metastatic CRC, and could facilitate a diagnosis of metastasis (AUC 0.766) (Liu et al., 2020). Furthermore, the level of exosomal miR-122 increased in CRC, especially in patients with liver metastasis, and its expression could help differentiate between patients with and without liver metastasis (AUC 0.81) (Sun et al., 2020).

Interestingly, other non-coding RNAs besides microRNA in liquid biopsy have emerged as potential biomarkers for metastatic CRC (Baassiri et al., 2020). Exosomal long non-coding RNA (lncRNA) CRNDE-h levels were significantly correlated with metastasis in CRC (Liu et al., 2016). Exosomal circular RNA (circRNA) hsa-circ0004771 was associated with distant metastasis (Pan et al., 2019). These findings suggest that circulating non-coding RNAs serve as novel potential diagnostic biomarkers for metastatic CRC.

#### Prognostic Biomarkers

With regard to the significance of providing prognostic and predictive information for cancer patients, liquid biopsy is promising as an indispensable clinical test for translational oncology. Increasingly, prognostic biomarkers for metastatic CRC patients have emerged with further research.

A study evaluating the prognostic value of ctDNA in 97 metastatic CRC patients showed that patients carrying more ctDNA had significantly decreased overall survival (OS), and the ctDNA fragmentation level was positively associated with shorter OS in the *KRAS/BRAF*-mutant cohort of patients, but not in the *KRAS/BRAF*-wild type cohort, indicating the roles of both qualitative and quantitative ctDNA analyses for prognostic assessment in metastatic CRC (El Messaoudi et al., 2016). This finding concurs with the conclusion of a meta-analysis that high pretreatment ctDNA levels correlated with shorter survival in patients with metastatic CRC (Spindler et al., 2017). A study of the prognostic value of pretreatment ctDNA in patients receiving first-line chemotherapy confirmed that increased ctDNA was associated with worse outcome in metastatic CRC patients (Hamfjord et al., 2019).

Multiple studies have confirmed the promising prognostic roles of baseline CTC for patients with metastatic CRC (Groot Koerkamp et al., 2013; Huang et al., 2015). In a prospective multicenter study comprising 430 metastatic CRC patients, Cohen et al. (2008) showed that patients with three or more baseline CTC had shorter median progression-free survival (PFS) (Cohen et al., 2008). Besides baseline CTC counts, the follow-up CTC levels persisted as strong predictors of PFS and OS during treatment for metastatic CRC patients. Importantly, the study further confirmed that the prognostic value of baseline CTC was unaffected by the characteristics of treatments or patients (Cohen et al., 2009). Besides CTC cell counts, the expression level of some genes in CTC may have prognostic effects. Ning et al. (2018) reported that the level of *Akt-2* expression in CTC could predict PFS in metastatic CRC patients, Patients with *Akt-2* expression in CTC had a significantly shorter PFS compared with those without *Akt-2* expression in CTC.

Several circulating microRNAs are associated with survival in CRC patients (Rapado-González et al., 2019). For metastatic CRC, high plasma miR-141 levels are significantly associated with poor survival in metastatic CRC patients (Cheng et al., 2011). Increased plasma miR-200a and plasma miR-122 levels correlated with decreased OS in metastatic CRC (Maierthaler et al., 2017). Additionally, high levels of extracellular vesicle miR-222 were associated with shorter OS in patients with metastatic CRC (de Miguel Pérez et al., 2020).

#### Therapy Response Monitoring and Guiding

Efficient markers of therapeutic response in patients with metastatic CRC are very important for guiding individualized treatment optimization strategies. The real-time abilities of liquid biopsy to monitor the dynamics of the cancer disease promisingly meet this demand.

In a prospective study involving 53 metastatic CRC patients receiving standard first-line chemotherapy, Tie et al. (2015) assessed ctDNA levels of patients at several timepoints, corresponding to pretreatment, 3 days post-treatment, and before the next treatment cycle. They found that largely decreased ctDNA levels (≥ 10-fold) before the next cycle were significantly associated with the trend of increased PFS in these patients, and concluded that the response to later radiologic treatment could be predicted by the early dynamics of ctDNA level during first-line chemotherapy (Tie et al., 2015). Garlan et al. (2017) found a similar conclusion among consecutive patients receiving first-line or second-line chemotherapy for metastatic CRC. For metastatic CRC patients diagnosed with wild-type *KRAS* who received a standard FOLFIRI-cetuximab treatment, Toledo et al. (2017) observed that continued wild-type circulating DNA status was associated with prolonged response to anti-EGFR therapy, whereas clinical deterioration could be predicted by the explosion of mutation events. For patients with *RAS/RAF* mutation and metastatic CRC who received standard first-line treatment, changes in ctDNA levels during the treatment were significantly correlated with low or high risk of disease progression (Thomsen et al., 2018). In patients with metastatic CRC referred for potentially resectable liver metastasis, a recent study showed that poorer postoperative survival was associated with persistently detectable preoperative ctDNA levels (Bidard et al., 2019). In a multicenter study that enrolled 47 patients with metastatic CRC with indications for resection, 93% (26/28) of patients with R0 resection did not have postoperative ctDNA, and the remaining R0 cases with detectable ctDNA had recurrence after 6 months (Benešová et al., 2019). These findings indicate that the early dynamics of ctDNA concentration would be valuable and efficient for monitoring therapeutic efficacy in patients with metastatic CRC.

Besides the ctDNA concentration, the status of ctDNA modifications, such as methylation, are potential biomarkers for monitoring therapy response in cancer patients. A study to identify corresponding methylated biomarkers of metastatic CRC in liquid biopsy to monitor the therapy response (Barault et al., 2018) showed that the dynamics of five genes (*EYA4*, *GRIA4*, *ITGA4*, *MAP3K14-AS1*, and *MSC*) methylation panel, without being affected by chemotherapy or targeted therapy, were significantly associated with the treatment response and PFS in patients with metastatic CRC (Barault et al., 2018). Another study including 123 patients with metastatic CRC treated with combination chemotherapy showed that the levels of neuropeptide Y (*NPY*) promoter methylation in ctDNA after one cycle of chemotherapy significantly correlated with survival (Thomsen et al., 2020). Of note, an additional advantage of detecting ctDNA methylation is a possible benefit of the liquid biopsy-based follow-up of cancer patients without information on mutation status.

Sastre et al. (2012) demonstrated that metastatic CRC patients with low baseline CTC counts had significantly better survival (both median PFS and OS) than those carrying a high CTC count at baseline. A similar survival bias was associated with the CTC counts after three cycles of chemotherapy plus bevacizumab (Sastre et al., 2012). In patients with wild-type *KRAS* and metastatic CRC who received monoclonal antibody treatment, changes in CTC levels from baseline to the first follow-up timepoint were significantly correlated with PFS (Souza et al., 2016). Likewise, the dynamics of gene expression in CTC and changes in CTC counts are promising predictors of the outcome of patients with metastatic CRC. A study determined the level of CTC markers, including tissue-specific and epithelial-to-mesenchymal transition genes (*GAPDH*, *VIL1*, *CLU*, *TIMP1*, *LOXL3*, and *ZEB2*), from 50 patients with metastatic CRC at baseline and at follow-up after treatment onset. The authors observed that patients with decreased CTC markers during therapy had significantly longer PFS and OS than those with elevated markers (Barbazán et al., 2014). Notably, treatment-resistant patients were identified by detecting the abovementioned CTC markers corresponding to the expression levels of a six-gene panel, although they were not diagnosed by routine imaging techniques (Barbazán et al., 2014).

Evidence indicates that circulating microRNAs representing non-coding RNAs are promising potential biomarkers for clinical evaluation in the treatment of patients with CRC (Rapado-González et al., 2019; Baassiri et al., 2020). A study of metastatic CRC patients with or without tumor response to 5-fluorouracil- and oxaliplatin-based chemotherapy identified three plasma microRNAs (miR-106a, miR-484, and miR-130b) that are highly expressed in chemoresistant patients. The study found that high pretreatment plasma levels of these miRNAs were significantly associated with shorter PFS (Kjersem et al., 2014). Another study concluded that levels of exosomal miR-21-5p, miR-1246, miR-1229-5p, and miR-96-5p were elevated in patients with chemoresistant advanced CRC. More importantly, the panel of exosomal these four microRNAs could predict (AUC 0.804) the chemotherapy resistance of individuals with advanced CRC (Jin et al., 2019). In patients with metastatic CRC treated with bevacizumab, basal plasma levels of miR-20b-5p, miR-29b-3p, and miR-155-5p correlated with PFS and OS, respectively. This suggests the potential outcome predictive roles of circulating basal miRNA levels in bevacizumab-treated metastatic CRC (Ulivi et al., 2018).

## Conclusion

Liquid biopsy, as a non-invasive and developing tool, could solve many limitations faced with conventional biopsies (Fernández-Lázaro et al., 2020). Studies have confirmed the promising roles of liquid biopsy in the clinical management of metastatic CRC. In the past decades, various biomarkers detected by liquid biopsy in metastatic CRC were found to be potentially useful in multiple areas, including auxiliary diagnosis of metastasis, prognosis prediction, and monitoring of therapy response. However, the practical clinical applications of these biomarkers in metastatic CRC, as well as other cancers, are very limited. One of the reasons for this is that most of the studies of the potential application of these biomarkers had small cohorts. Therefore, large, multicenter, and prospective clinical validation trials are needed for the implementation of these liquid biopsy-based biomarkers in clinical scenarios. Moreover, the standardization of the liquid biopsy process, for sample collection and subsequent analysis, is necessary in the clinical setting.

## Author Contributions

WG designed the study. WG, YC, JY, and CZ collected the data and wrote the manuscript. SH, HZ, and YS reviewed the manuscript. All authors contributed to the article and approved the submitted version.

## Conflict of Interest

The authors declare that the research was conducted in the absence of any commercial or financial relationships that could be construed as a potential conflict of interest.
